# Visual instrumental learning in blindsight monkeys

**DOI:** 10.1038/s41598-021-94192-7

**Published:** 2021-07-20

**Authors:** Rikako Kato, Abdelhafid Zeghbib, Peter Redgrave, Tadashi Isa

**Affiliations:** 1 Department of Developmental Physiology, National Institute for Physiological Sciences, Okazaki, Japan; 2 Department of Neuroscience, Graduate School of Medicine, Kyoto University, Yoshida-konoe-cho, Sakyo-ku, Kyoto, 606-8501 Japan; 3 Department of Psychology, University of Sheffield, Sheffield, UK; 4 Department of Life Science, The Graduate University for Advanced Studies (SOKENDAI), Hayama, Japan; 5 Human Brain Research Center, Graduate School of Medicine, Kyoto University, Kyoto, Japan; 6 Institute for the Advanced Study of Human Biology (WPI-ASHBi), Kyoto University, Yoshida-konoe-cho, Sakyo-ku, Kyoto, 606-8501 Japan

**Keywords:** Operant learning, Consciousness, Visual system

## Abstract

Blindsight is the residual visuo-motor ability without subjective awareness observed after lesions of the primary visual cortex (V1). Various visual functions are retained, however, instrumental visual associative learning remains to be investigated. Here we examined the secondary reinforcing properties of visual cues presented to the hemianopic field of macaque monkeys with unilateral V1 lesions. Our aim was to test the potential role of visual pathways bypassing V1 in reinforcing visual instrumental learning. When learning the location of a hidden area in an oculomotor search task, conditioned visual cues presented to the lesion-affected hemifield operated as an effective secondary reinforcer. We noted that not only the hidden area location, but also the vector of the saccade entering the target area was reinforced. Importantly, when the visual reinforcement signal was presented in the lesion-affected field, the monkeys continued searching, as opposed to stopping when the cue was presented in the intact field. This suggests the monkeys were less confident that the target location had been discovered when the reinforcement cue was presented in the affected field. These results indicate that the visual signals mediated by the residual visual pathways after V1 lesions can access fundamental reinforcement mechanisms but with impaired visual awareness.

## Introduction

Blindsight is the residual visuo-motor ability observed in some patients with damage to the primary visual cortex (V1)^1–3^. Despite profound visual impairment, a range of visual functions are preserved both in humans^4,5^ and in a nonhuman primate model of blindsight that has experimental unilateral V1 lesions^6–13^. However, apart from a study with a human blindsight patient that investigated the classically conditioned startle reflex^14^, visual associative learning competences in blindsight subjects remain to be investigated.

Associative learning is a fundamental aspect of brain function that animals use to modify their behavior in natural environments. When confronted with stimuli that predict reward or punishment, or the need to understand action-outcome relationships, associative learning permits animals to acquire novel adaptive responses. Two forms of associative learning are recognized: (1) Pavlovian or classical conditioning that associates predictive (conditioned) stimuli (CS) with (unconditioned) rewards or punishment (UCS)^15^. After training, the CS predictor elicits anticipatory (conditioned) responses (CR). (2) Instrumental or operant conditioning associates behavioral output with contingent outcomes. We recently tested Pavlovian conditioning in the hemi-blindsight monkeys and showed a reliable conditioned response when the CS was presented to the lesion-affected visual field. We were able to clarify the subcortical neural systems underlying this performance^16^.

Instead of associating two sensory events in Pavlovian conditioning, instrumental/operant conditioning permits animals to learn novel responses that are instrumental in acquiring rewards and avoiding punishments^17,18^. However, when a neutral stimulus is classically conditioned to reward/punishment, it can act as an effective secondary reinforcer of instrumental conditioning. In this study, to test whether instrumental learning is preserved in blindsight subjects, we used a conditioned visual stimulus presented in the lesion-affected field to reinforce the responses required to discover the location of a hidden area on blank screen (an area without any visual indication). In monkeys with unilateral V1 lesions saccadic eye movements that moved their gaze into the hidden target area were reinforced (“hidden area search task”). This task was similar to that used by Chukoskie et al.^19^ and well characterized by a reinforcement-learning model that maintained and updated a reward map of locations. The present study is also important in the context of an ongoing debate concerning the role of conscious awareness in associative learning^20–24^. Aspects of our experiment, in particular the seeming unawareness of animals when the secondary reinforcing stimuli was presented to the lesion affected visual field, provides further evidence that novel instrumental responses can be acquired in the absence of normal visual awareness.

## Results

### Unilateral V1 lesion

**Figure 1:**
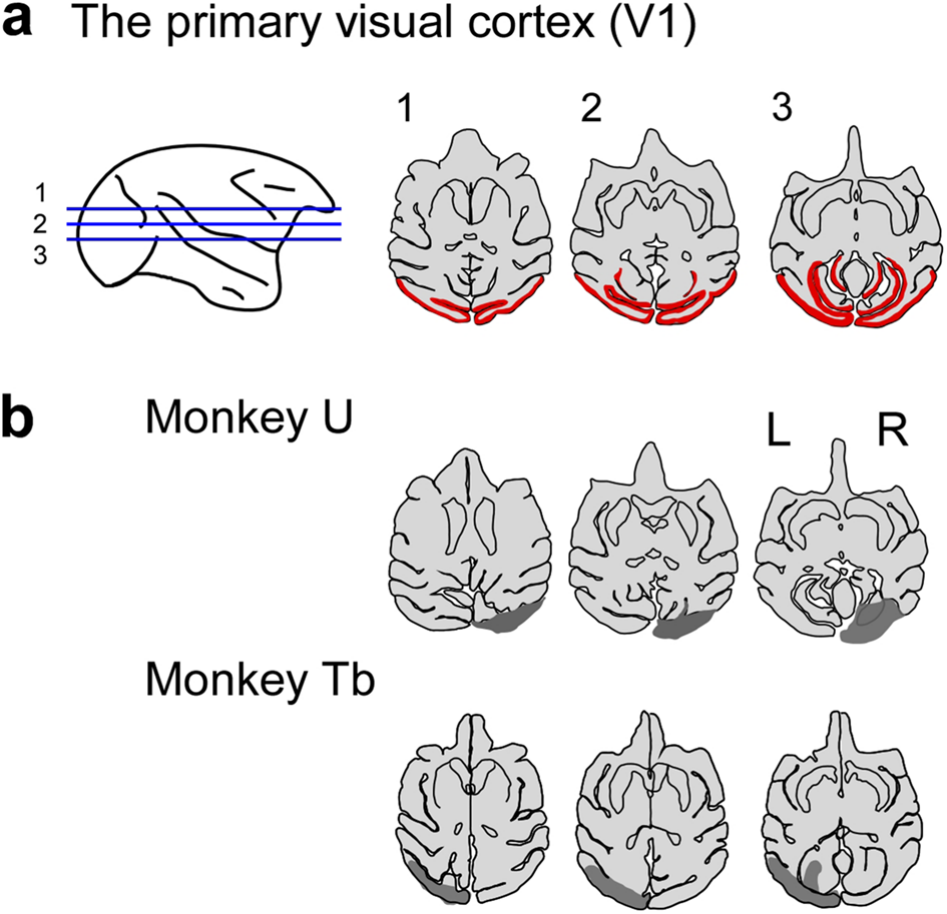
Unilateral V1 lesion. Extent of unilateral V1 lesion in monkeys U, Tb. (**a**) and (**b**) MR images of horizontal sections of the monkey’s brain were traced and the extent of lesion was drawn on the traces. (**a**) The bilateral V1 is shown in red on the trace of monkey U before lesion. (**b**) The lesion sites (gray area) of monkeys U and Tb on the traces. Lesion sites for monkey U and monkey Tb were described in previous papers^8,12^. However, the horizontal sections of monkey U and monkey Tb in (**b**) were obtained from more recent MR images compared to those published in the previous literature.

The lesion sites extended over the opercular surface of the striate cortex and the medial area in the Calcarine Sulcus (Fig. 1a, b). Only the foveal site located in the ventrolateral part of the opercular surface (visual field for eccentricity 0°–1.0°) was intended to be left intact. Sensitivities to luminance contrast in the contralesional visual field after the V1 lesion were impaired as presented in the deficit map in Supplementary information (SFig. 1).

### Performances in the hidden area search task

**Figure 2:**
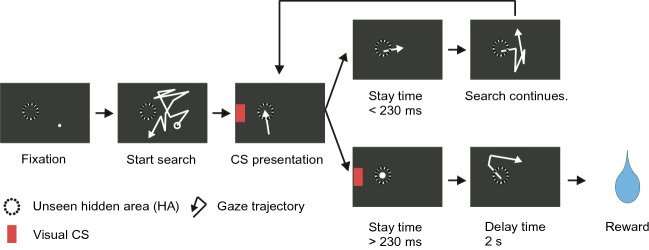
Hidden target area search task. Sequence of trial events in the search task. In each trial the offset of randomly located fixation point signaled that the search for the unseen hidden area (HA) could begin. When the monkey’s gaze entered the HA, a reinforcing visual CS was presented.

Using saccadic eye movements, the animals were required to search for a hidden area (HA) located within the blank monitor screen (Fig. 2). If the monkey’s gaze moved to and remained in the HA for 30 ms, the reinforcing visual CS was presented at the left or right edge of the screen. In this way it would presented either to the lesion-affected, or intact visual hemifield. Taking into account the normal time to transition between saccades, the monkeys were required to maintain their gaze within the HA for a stay time period of > 230 ms. Then, a pause in the HA that satisfied this criterion caused a drop of juice reward to be delivered with a delay of 2.0 s. After the location of HA has been judged to be learned (see the Method section), it was repositioned to a new location and a new learning session initiated. Prior to the formal testing of instrumental conditioning, the monkeys were trained in sessions of the hidden area search task where large target areas ensured that the Pavlovian association between the visual cues and the juice reward was firmly established. This procedure was conducted to confer secondary reinforcing properties to the visual cues.

In 2 monkeys, a total of 58 learning sessions were conducted, each of which contained  > 100 trials (26 sessions when the CS was presented to the intact side, and 32 sessions for CS in the lesion-affected side). Additional criteria were used to select 35 sessions on which a complete set of analyses were performed (20 sessions for CS in the intact side and 15 sessions for CS in the lesion-affected side) (See Supplementary information).

#### CS in the intact field

**Figure 3:**
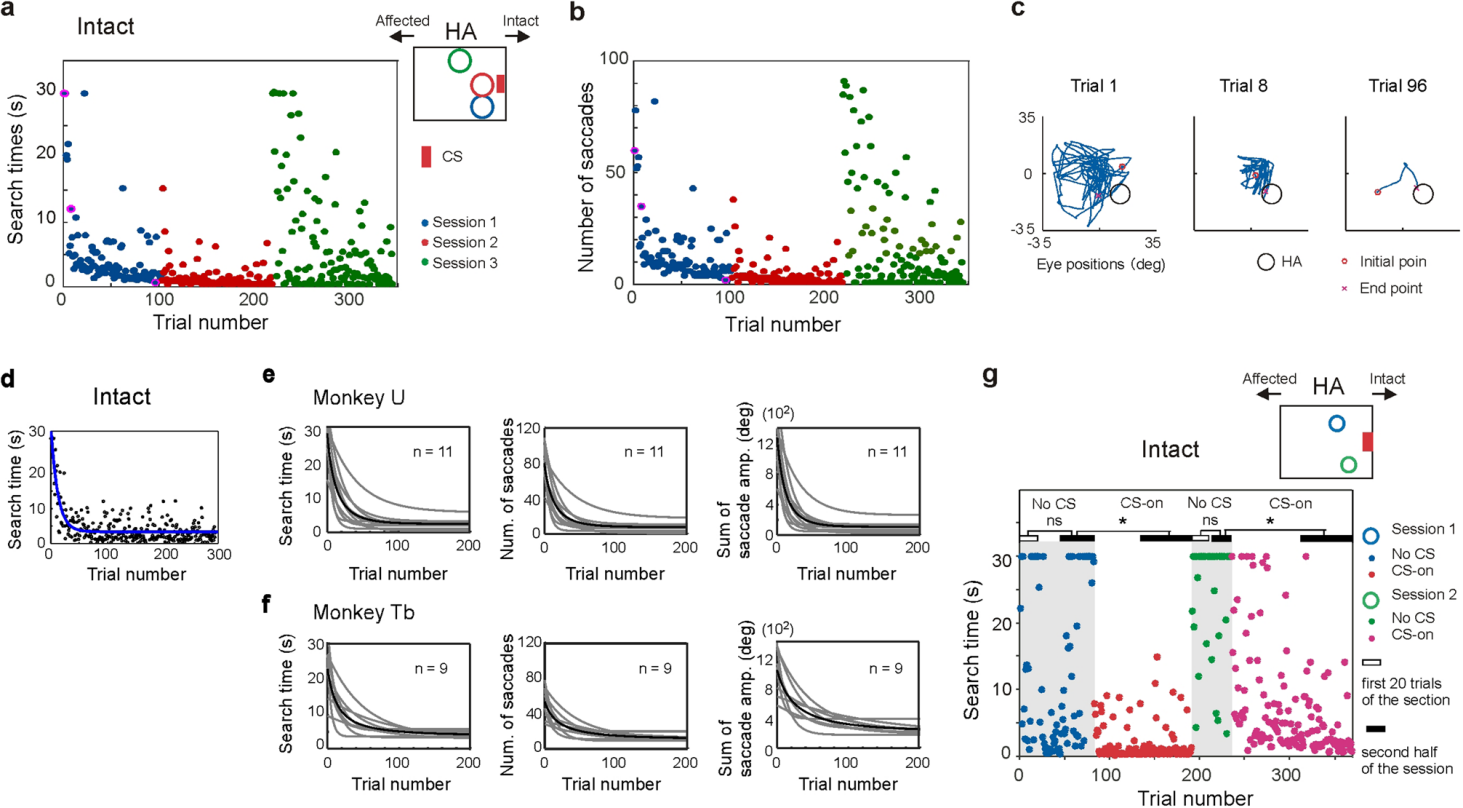
Instrumental learning with CS in the intact visual field. (**a**) and (**b**) Examples of reduction in search time (**a**) and number of saccades (**b**) when the CS was presented in intact field. Search time and number of saccades for each trial was plotted against the trial number. HA position was altered in each session. (**c**) Eye position trajectories for three trials (trial 1, trial 8, trial 96) of session 1. The three trials are labeled by magenta circle in (**a**) and (**b**). (**d**) The optimally fitting decaying exponential curves (blue line) are superimposed on an example dataset for which the search time are plotted against the trial number in the session. The CS was presented in the intact visual hemifield. (**e**) and (**f**) Population data that characterize the learning of HA location in the instrumental conditioning task. (**e**) Population data from monkey U. Decaying exponential fitting curves from sessions in the intact condition were superimposed. Means of the curves are indicated as thick black lines. (**f**) The population data from monkey Tb, the same arrangement as (**e**). (**g**) No CS condition. Learning failed to occur in the No CS conditions (grey hatched area) but was reinstated when the CS was presented to the intact field. *Wilcoxon rank sum test, p < 0.05.

We started by testing instrumental learning when the CS was presented in the intact visual field (Fig. 3). Within an experimental session the early trials were characterized by the monkeys’ shifting their gaze widely across the visual field searching for the HA (Fig. 3a–c). However, with repeated trials, the time taken to move to the HA’s location became progressively shorter and in parallel the number of saccades used to locate the HA became smaller (Fig. 3a, b, blue trials). When the location of the current HA had been learnt, the monkeys used just 2–3 saccades, expressed in 0.5–2 s, to shift their gaze from the initial fixation location to the HA. At this point the HA was then moved to different location and a new learning session was initiated. Unsurprisingly, when the new HA was close to that in the preceding session, the new location was learned quickly (Fig. 3a, b, red trials). On the other hand, in the next session which the new HA was moved to a relatively distant location, the time taken to discover its location was considerably longer. However, successful trials progressively increased and the time taken to find the new location systematically decreased (Fig. 3a, b, green trials). This observation suggests that the animals’ search strategy was influenced by recent past experience where the time was spent searching near the previous HA location to get a cue associated with a reward. Formal analysis of all 20 sessions involving CS presentation in the intact visual field revealed that search times were significantly shorter during the second half of the session compared with those during the first 20 trials (Wilcoxon rank sum test, p < 0.05, in 11/11 sessions in monkey U and 9/9 sessions in monkey Tb, all statistical tests were two-tailed.). In Fig. 3d, fitting curves for a session estimated by the decaying exponential function were superimposed on the data points for the search time and evaluated by the distribution of residuals. For both subjects, the search time, the number of saccades made to locate the HA, and the sum of saccade amplitudes over trials were fitted by decaying exponential curves (Fig. 3e, f). The parameters used to fit the exponential curve to the data in each session are indicated in STable 1 and 2. These figures reveal the range of time courses over which learning occurred. The range of Trial index (The trial number which coming down from the point of “− 50 ms/trial” on the fitted curve) were 24–104 in monkey U and 23–115 in monkey Tb. Thus, in each session reliable instrumental conditioning was demonstrated.

To rule out the possibility that the delayed presentation of the primary reinforcer (juice) was responsible for the instrumental learning, we instituted a blocks of trials in some sessions where everything was held constant except that CS presentation was omitted (‘No CS’ condition: see the Method section). Under these conditions the animals failed to learn the HA location (light-grey background in Fig. 3g). A reliable decrease in search time between trials in the second half of the block compared with the first 20 trials was not observed (Wilcoxon rank sum test, p > 0.05, in 2/2 sessions in monkey U). However, when the CS presentation was reinstated and the HA location unaltered the animals were able to discover the location of the HA (white background in Fig. 3g). Thus, search times significantly decreased during the second half of the block with ‘CS’ condition compared with those during the second half of the preceding block of the ‘No CS’ condition. (Wilcoxon rank sum test, p < 0.05, in 2/2 sessions in monkey U). These observations indicate that, in our task, the visual CS that appeared 30 ms after the entrance of gaze into the HA, rather than the primary juice reward which arrived 2 s later, was the critical reinforcer that enabled instrumental learning of HA locations.

#### CS in the affected field

**Figure 4:**
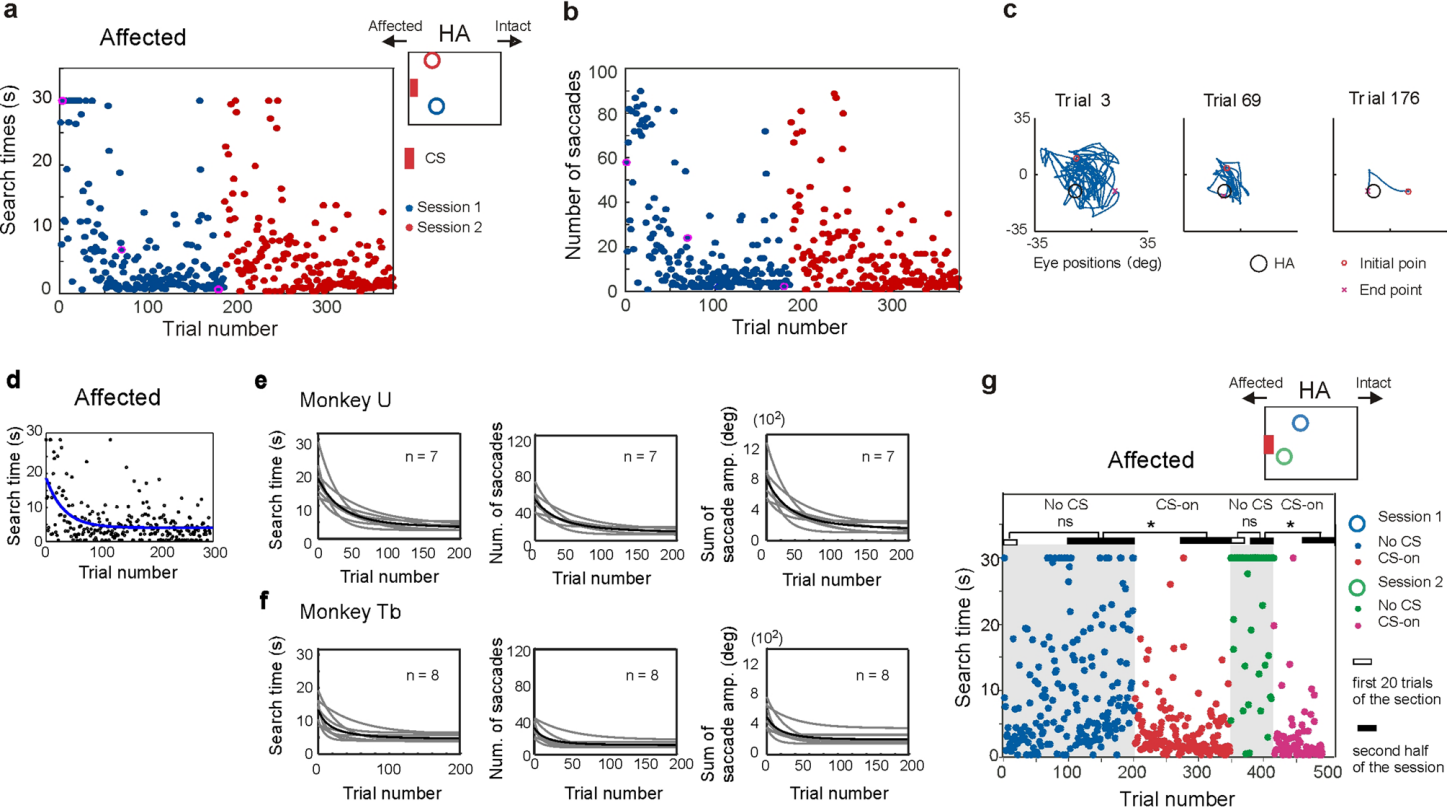
Instrumental learning with CS in the affected visual field. (**a**) and (**b**) Examples of reduction in search time (**a**) and number of saccades (**b**) when the CS was presented in the affected field. Search time and number of saccades for each trial was plotted against the trial number. HA position was altered in each session. (**c**) Eye position trajectories for three trials (trial 3, trial 69, trial 176) of session 1. The three trials are labeled by magenta circle in (**a**) and (**b**). (**d**) The optimally fitting decaying exponential curves (blue line) are superimposed on an example dataset for which the search time are plotted against the trial number in the session. (**e**) and (**f**) Population data that characterize the learning of HA location in the instrumental conditioning task. (**e**) Population data from monkey U. Decaying exponential fitting curves from sessions in the affected condition were superimposed. Means of the curves are indicated as thick black lines. (**f**) The population data from monkey Tb, the same arrangement as (**e**). (**g**) No CS condition. Learning failed to occur in the No CS conditions (grey hatched area) but was reinstated when the CS was presented to the affected field. *Wilcoxon rank sum test, p < 0.05.

The main purpose of the study was to test whether a visual CS presented to the lesion-affected visual field was also able to reinforce the acquisition of novel instrumental behavior. In sessions when the visual CS was presented to the lesion-affected visual field the observed learning was qualitatively similar to when it was presented to the intact visual field (Fig. 4a–c). In most sessions, search times significantly decreased during the second half of the session compared with those during the first 20 trials (Wilcoxon rank sum test, p < 0.05, in 6/7sessions in monkey U and 6/8 sessions in monkey Tb). Again, the search time, the number of saccades and the sum of saccade amplitudes over trials were all reliably fitted by decaying exponential curves (Fig. 4d–f and STable 1 and 3). The range of Trial index were 49–97 in monkey U and 23–73 in monkey Tb. In the present study, it was not possible to conduct quantitative comparisons of the learning efficiency between CS presentation in intact vs lesion-affected visual fields because the task difficulty was not balanced between the two conditions (see Supplemental information: Limitation of this study).

The condition in which the CS was omitted was also tested for some trials in sessions where the CS was being presented to the lesion affected visual field. Again, under these conditions the animals failed to learn the HA location (light-grey background in Fig. 4g, Wilcoxon rank sum test, p > 0.05, in 2/2 sessions in monkey U and in 2/2 sessions in monkey Tb). After the visual CS was re-instated, the animals were again able to discover the location of the HA (white background in Fig. 4g, Wilcoxon rank sum test, p < 0.05, in 2/2 sessions in monkey U and in 2/2 sessions in monkey Tb). These observations replicate the finding that for instrumental learning to occur, the near immediate visual CS presented to the lesion-affected field in this condition is also essential, rather than the delayed juice reinforcement.

Taken together, these data show that for both subjects the instrumental learning required to locate an unseen area could be successfully accomplished when a secondary reinforcing visual CS was presented either to their intact or to their lesion-affected visual field. Importantly the features of the learning performance in either case was qualitatively comparable.

### Targets of reinforcement

**Figure 5:**
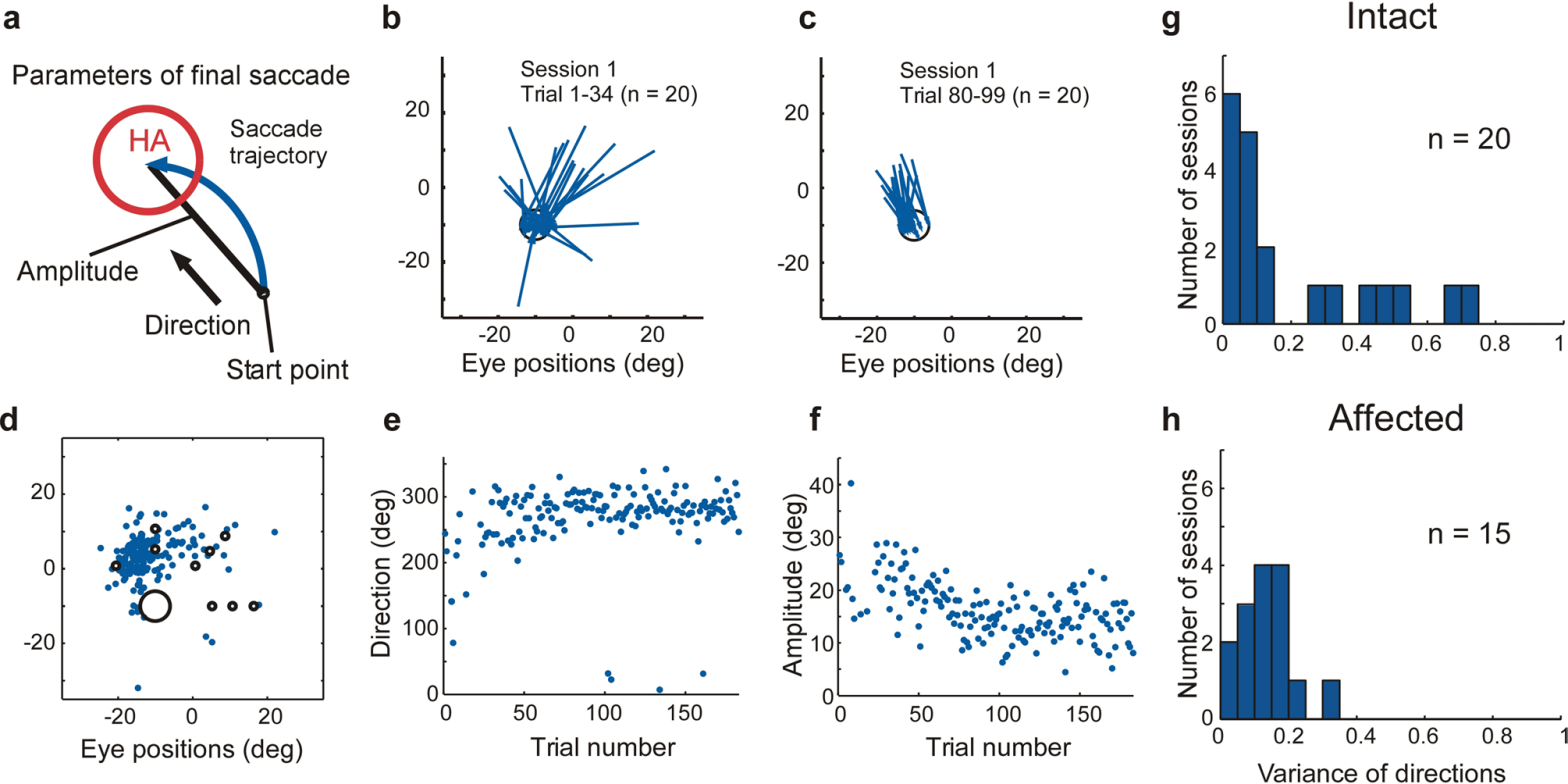
Learning the matrix of the final saccades that preceded CS presentation. (**a**) Schematic illustration of a final saccade capturing the HA and its parameters. (**b**) and (**c**) Vectors of final saccades when the CS was presented in the lesion-affected field. The final saccades of the 20 successful trials among the trial No. 1–34 (**b**) and trial No. 80–99 (**c**) of a session which is the first session in Fig. 4a, b. A final saccade was characterized by three parameters [start point (**d**), direction (**e**) and amplitude (**f**)] of the vector from its onset location to its offset in the HA (**a**). (**d**)–(**f**) Saccade parameters in a session, shown in (**b**) and (**c**). Start point (**d**), direction (**e**) and amplitude (**f**) of the final saccades. (**g**) and (**h**) Distribution of variance of direction of the final saccades in multiple sessions. (**g**) CS in the intact field and (**h**) lesion-affected field.

We next addressed the issue of what aspects of saccadic eye movements in our search task were being reinforced by the visual CS. To force the animal to learn the HA location in a representation in the non-retinocentric coordinate space (either head-centered or allocentric), each trial the search for the HA was started from a different randomly assigned location. This prevented a retinocentric solution based on learning a vector of eye movements from a fixed starting position. In our task, optimal performance in the non-retinocentric coordinate frame would be for the monkey to make a single saccade from the initial, randomly determined start location directly to the HA. However, in practice, this response rarely occurred, even after the location of the HA was well known (see trial 96 in Fig. 3c and trial 176 in Fig. 4c). We found that the final eye movements entering the HA (Fig. 5a) tended to converge on one or two vectors in each session (i.e. the amplitude and direction of final saccades became increasingly consistent; Fig. 5). For example, in the session shown in Fig. 5b–f, the preponderance of final saccades entered the HA from above (median saccade direction = 282°). The ‘variance in direction’ in Fig. 5g, h was defined as V = 1 − R (R: length of mean saccade direction vector, 0 ≤ V ≤ 1, see Supplementary information). Here, a value of 1 indicates a uniform dispersion from all possible directions, while a value of 0 would represent entry from a single direction. The variance of saccade direction in the case of Fig. 5e was 0.15 and in a majority of cases (13 out of 20 in the intact field and 13 out of 15 in the affected field), the values were < 0.2 (Fig. 5g, h). In both conditions with CS in intact and affected visual field, standard deviation (SD) of the amplitude of the final saccades was mostly under 6° (18 out of 20 in the intact field and 12 out of 15 in the affected field) and SDs of start points along the horizontal and vertical axes were mostly less than 10° (19 out of 20 in the intact field and 14 out of 15 in the affected field). Thus, it appeared that the reinforcement afforded by the visual CS caused the non-retinocentric (either head-centered or allocentric) neural system to learn the start location of the final saccade and the retinocentric neural system to learn the vector from this location to the HA. This sub-optimal performance, that is learning the non-retinocentric location of the final saccade start position and its retinocentric vector to the HA, can both be considered an example of ‘superstitious’ reinforcement learning^25,26^. Importantly these observations suggest that reinforcing effect of the CS in our task is broadcast to systems capable of learning in both non-retinocentric and retinocentric coordinate frames.

### Behavior after visual CS presentation

**Figure 6:**
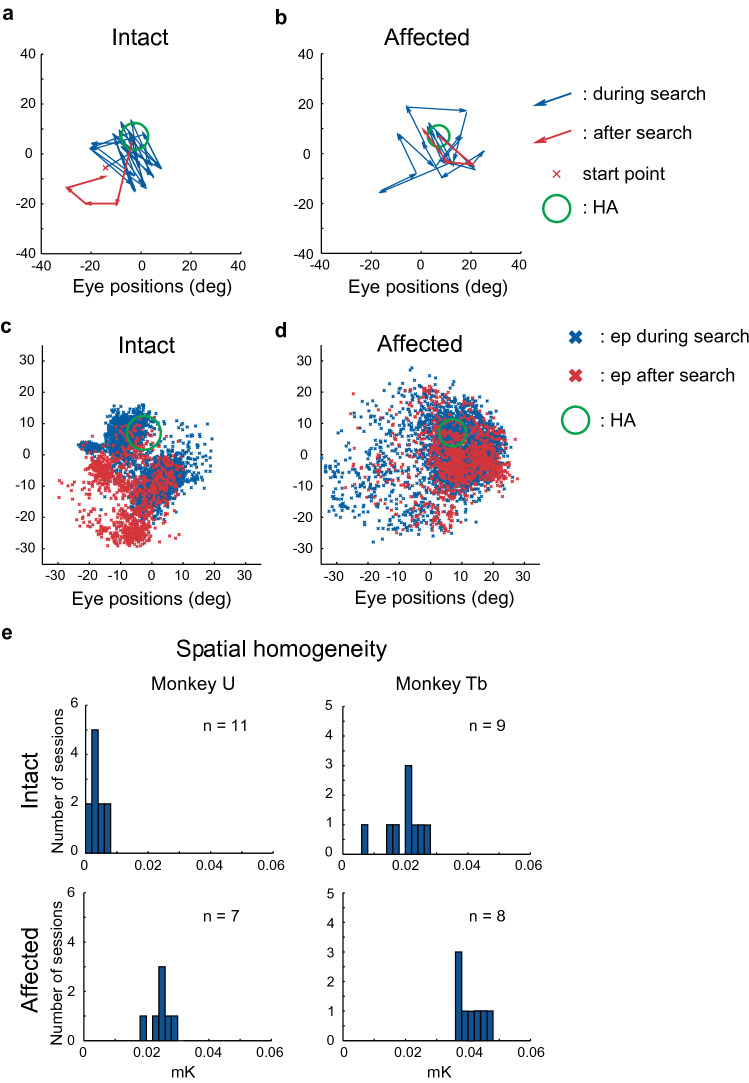
Patterns of eye movements differed after CS presentation to the intact and lesion-affect visual fields. (**a**)–(**d**) During a single search trial, saccades before (blue lines) and after (red lines) CS presentation to the intact (**a**) and lesion-affected (**b**) visual fields. In a whole session, the distributions of saccade end points (ep) before (blue) and after (red) CS presentation to the intact (**c**) and lesion-affect (**d**) visual fields. (**e**) Spatial homogeneity of distributions of saccade end points in each session calculated by modified Rippley’s K function between before and after CS presentation to the intact and lesion-affected visual fields (mK).

Some of the data from our study is relevant to the ongoing debates concerning the seeming lack of subjective awareness of visual cues that can guide movement in ‘blindsight’^1–3^, and have become associated with primary reward or threat^20–24^. As a contribution to the problem of subjective awareness we analyzed the pattern of eye movements after the visual CS had been presented in our instrumental learning task. The simple idea was that when you find something you are looking for, you stop looking—but only if you are confident that you have found it. Thus, in our study when the visual CS was presented in the intact field, the monkeys appeared to stop searching and move their gaze away from the HA location (Fig. 6a). Consequently, the distribution of saccadic end points after presentation of the visual CS differed significantly from those during search (Fig. 6c). This suggests that the monkeys were subjectively aware and confident that the visual signal indicating that the HA location had been found, had been presented. At this point in the trial, it would be obvious that further searching was no longer necessary, and that the predicted primary reward would shortly be delivered. In contrast, when the visual CS was presented to the lesion-affected field, the monkeys appeared to continue searching in and around the location of the HA (Fig. 6b, d). By inference, this suggests that the monkeys were either unaware, or subjectively uncertain, that the visual CS had been presented, and therefore continued to search. The statistical reliability of these observations was evaluated by applying modified Ripley's *K* functions^27^. These statistics assessed the extent to which the distributions of saccade end points before and after the CS presentation over sessions were similar, or different. In both monkeys, values of spatial homogeneity were significantly higher in the condition where the visual CS was presented to the affected field (Fig. 6e; Wilcoxon rank-sum test, p < 0.001). These observations could represent a novel measure of potential subjective awareness in non-human primates that could complement the yes/no task used by Cowey and Stoerig^28^ and in our previous study^29^. Moreover, the results also suggest that the neural systems responsible for instrumental associative learning in our search task can operate effectively even when the monkey is seemingly unaware or uncertain that the reinforcing visual CS had been presented.

## Discussion

The purpose of the present investigation was to test whether visual systems, other than the primary visual cortex, can use the appearance of secondary reinforcing visual cues for the acquisition of novel instrumental behavior. To achieve this, we conducted experiments in monkeys with unilateral experimental lesions of V1. The animals were required to use saccadic eye-movements to discover the location of a hidden target area. The main findings of the study were: (1) The monkeys were able to discover the location of the hidden area, even when the secondary reinforcing stimulus was presented in V1 lesion-affected visual field. (2) Sub-optimal performance in the oculomotor search task was observed where animals learned the start position and vector of the final saccade that entered the target zone, independently of the different locations from which the search was started. (3) The pattern of oculomotor behavior after the secondary visual reinforcing signal was reliably different when it was presented in the V1 lesion-affected visual field, compared with when it was presented in the intact visual field. Below, we will discuss these findings in turn.

A peculiar feature of the present “hidden area search task” is that the monkeys had to move their gaze around a blank screen without any cue stimulus to learn the location of an unseen hidden area (HA). After entry to the HA, the entry was signaled by a reward-predicting visual CS presented either in the intact or V1 lesion-affected visual field. We found that the animals’ ability to discover the location of the target zone was retained when the CS was subsequently presented in the lesion-affected visual field. This suggests that visual systems in the brain, other than primary visual cortex, have access to the reinforcement mechanisms responsible for instrumental conditioning. The visual competences demonstrated after damage to V1 have been associated with neural processing in the retino-recipient midbrain superior colliculus (SC), which is similarly evolutionary ancient^6,7,12^, and/or the SC-geniculo-extrastriate cortical pathway^30^. In addition, our recent study showed that the SC-pulvinar pathway can mediate the signal for visuo-motor transformations in blindsight monkeys^13^. In the present study, we used a simple luminance-based shape as the CS. Therefore, the most direct route whereby CS-related information could gain access to reinforcement mechanisms in the basal ganglia is likely to have been via the retino-tecto-nigral projections^16,31–33^. Although, other pathways through the thalamus (pulvinar and/or LGN)-other brain area-nigra pathway) could also provide access to the basal ganglia^30,34,35^.

Furthermore, it has been claimed that a critical substrate for instrumental learning is the modulation of cortico-striatal and thalamo-striatal synaptic transmission by unpredicted sensory-evoked phasic activity of ventral midbrain dopamine neurones^36^. In the present study, we would predict that *presentation of our secondary reinforcing visual CS* should induce a short-latency phasic dopamine response, irrespective of whether it was presented in the intact or lesion-affected visual field. Moreover, that the consequent release of dopamine into basal ganglia nuclei should reinforce the immediately preceding neural signals that caused the action responsible for the eyes to move to the HA. Such plastic changes in the neural circuits accompanying the instrumental learning shown in the present study should be the subject of future studies.

When the location of the HA had been discovered a sub-optimal strategy for directing gaze to the HA was frequently observed. Rather than simply move their gaze from randomly located start positions directly to the HA, the monkeys’ eye-movements typically went first to a location close to the HA before entering it with the final saccade (Fig. 3c trial 96; Fig. 4c trial 176). How should these observations be interpreted? First, it is important to recognize that actions are multidimensional and, depending on the task, the different action dimensions can be learned independently^19,37,38^*.* Thus, a subject may have to learn *where* to perform an action in allocentric coordinate space, *what* action to do when they get there (typically in egocentric space), *how* to perform the action (fast/slow, forceful/gently) and *when* to perform it. For any particular task, it is likely that each of these dimensions has to be separately learned in specialized networks within the brain^39–41^. Consequently, the simplest explanation of the present data is that the CS-evoked reinforcement signal was broadcast widely to both egocentric and allocentric neural representations capable of guiding the animal’s gaze^42^. Thus, it is likely that *all* movement dimensions of the final saccade that triggered the CS—its initial start position in allocentric space *and* its vector (direction and amplitude) in egocentric space—were reinforced, rather than just the end-position of the movement in the HA in allocentric space. It is well known that saccade-related signals in many of the saccade-related regions such as frontal eye field, basal ganglia and superior colliculus are basically encoded in the retinocentric coordinates^43–46^. These saccade-related regions are likely to have been activated in the present task, and therefore responsible for learning the various retinocentric (egocentric) parameters of the final saccade. Rather than learning the movement that would take the gaze directly from the start position to the HA, learning the location of the final saccade’s start position and subsequent vector to the HA can be thought of as examples of ‘superstitious’ behavior^25^. More generally, the concept of a widely broadcast reinforcement signal acting on spatially distributed^42^, but differentially relevant dimensions of action (‘where’, ‘what’, ‘when’ and ‘how’) could provide a unifying explanation for often observed sub-optimal (superstitious) behavioural performance^47^. Moreover, if the first saccade from the FP misses the HA, it would become difficult to reinforce the direct saccade to HA from the start position of the trial by a later CS presentation. It would take time to reinforce all direct movements from 9 to 12 possible start positions in each session. Instead, it was always the final saccades in the trial that were primarily reinforced by the CS. Then, sub-optimal behavior (repeating previous final saccade to the HA) might come into existence as a process of the learning.

In the present study, the visual CS signaled that the location of the hidden area had been discovered. When the CS was presented in the intact visual field, the monkeys appeared to stop searching, thereby indicating they were aware that the HA had been discovered. In contrast, when it was presented to the V1 lesion-affected field, the animals appeared to continue searching in and around the HA. By inference, this suggests that the V1-lesioned monkeys were subjectively unaware or unsure that the visual CS had been presented. This discovery could usefully inform the ongoing debate about the necessity of subjective awareness of reinforcement cues in associative learning. The loss of visual awareness in an animal model of ‘blindsight’ was first reported by Cowey and Stoerig^28^, who showed that monkeys with V1 lesions exhibited near-zero performance in reporting the appearance of visual target in a “Yes–No” behavioral paradigm. The shortcomings of the original Cowey and Stoerig study^48^ were more recently addressed in a report from our laboratory^29^. Our results confirmed that the sensitivity of target detection analyzed with signal detection theory in Yes–No task was significantly impaired relative to that in the forced-choice task. Together these findings are consistent with the performance of the famous blindsight patient G.Y. who also showed differential visual competences in Yes/No and forced choice tasks, while maintaining a loss of visual awareness to the presentation of static visual stimuli^49^. However, methodological constraints unavoidably surround investigations of subjective awareness in animal associative learning. However, by showing the monkeys continued to search after presentation of a visual CS that had effectively reinforced discovery of a hidden target, the present study offers a complementary paradigm to assess the subjective awareness of visual stimuli in hemi-blindsight non-human primates, and possibly also in human blindsight patients.

## Methods

### Subjects

The present study used two adult Japanese monkeys (hereafter Monkeys U and Tb; macaca fuscata; both female, body weight 5.1–5.5 kg). Having taken part in these previous investigations^8,10,12^, the present experiments were initiated 44–46 and 94–96 months after the V1 lesions in monkey U and monkey Tb, respectively. The experiments were conducted in the National Institute for Physiological Sciences, which is a part of the National Institutes of Natural Sciences. All experimental procedures were performed in accordance with the National Institutes of Health Guidelines for the Care and Use of Laboratory Animals and Basic Policies for the Conduct of Animals Experiments in Research Institutions by MEXT, Japan, and approved by the Committee for Animal Experiments at the National Institutes of Natural Sciences, Japan. The monkeys were acclimatized to the experimental locations over two months. Details of the procedures for training and surgery of the monkeys have been described in detail in previous reports^8,12^. In brief, under anesthesia with 1.0–1.5% isoflurane inhalation, the monkeys were implanted with scleral search coils for eye position measurement, and head post holders in aseptic conditions. The monkeys were allowed to recover for more than 2 weeks before starting pre-lesion training and then were trained to perform a visually guided saccade task. During the behavioral task, the monkey’s head was stabilized by attaching the implanted head holder to a frame. The animals were required to move their eyes from a fixation point (FP) to localize a visual stimulus which appeared on a monitor positioned 42 cm in front of their face. The monkeys’ eye movements were measured by search coil technique (sampling rate: 1 kHz)^50^. Stimulus presentation and data collection were executed by a real-time experimental control system (Tempo for Windows, Reflective Computing; http://reflectivecomputing.com/).

Under isoflurane anesthesia, the right V1 of monkey U, and left V1 of monkey Tb were surgically removed by aspiration (suction tube tip diameter; 1–1.5 mm).

### Hidden area search task

This task was used in the current study to determine whether the animals could acquire instrumental responses reinforced by a conditioned visual stimulus. For this purpose, we used an oculomotor version of a joy-stick task developed for rats^38^. Specifically, using their eye-movements the animals had to search on a monitor screen for a hidden, experimenter defined hidden area (HA) (Fig. 2). A similar visual search task for hidden target area has been reported with human subjects^19^. The advantage of this task is that it allows for repeated assessments of instrumental learning. Once the location of one hidden area has been discovered, the hidden target zone can be repositioned and the process of learning a new location can begin. Prior to the formal testing of instrumental conditioning, the monkeys were trained in sessions of the hidden area search task where large target areas ensured that the Pavlovian association between the visual cues and the juice reward was firmly established. This procedure was conducted to confer secondary reinforcing properties to the visual cues.

Each trial was started by requiring the monkey to fixate a fixation point (FP) for 500 ms. The FP appeared randomly at a one of 9–12 possible positions arranged around a circular unseen HA. This ensured that the animals had to learn a location on the monitor in allocentric coordinates, un-confounded by the possibility of learning an egocentric movement. After the initial fixation period, the FP was extinguished, which signaled to the monkey to begin searching for the HA. The positions of the HA were selected in a pseudorandom manner within 27.8° square on the screen. The size of target areas was adjusted between 8° and 15° in diameter. To trigger the reinforcing visual CS the monkey’s gaze had to remain in the HA for > 30 ms. This was to exclude saccades passing over the HA without a pause. Whenever the HA was located, the visual CS [rectangle, 1.1° horizontal and 2.2° vertical, luminance contrast: Michelson contrast 0.88–0.94 (Weber contrast 14.7–31.3)] was presented at the left or right edge of the screen (25 deg in eccentricity from the center of monitor), corresponding to the lesion-affected or intact visual hemifield. We did not set the HA on the other side of monitor relative to the CS position when the CS was presented in the affected visual field. As shown in previous our researches^8,12^, the extent of lesion covered 5°–25° or 1°–25° in eccentricity of the contralesional visual hemifield in our V1-lesioned monkeys because more peripheral part of the visual field is represented in the deepest part of the Calcarine Sulcus and we avoided removing the tissue representing the visual field of over 30° in eccentricity during the surgery. Therefore, had we placed the HA on the other side of the monitor, the CS could have fallen out of the visual field affected by the V1 lesion. After presentation of the visual CS, the monkeys were required to maintain their gaze within the HA for another 200 ms to assure that the HA location had been discovered. Thus, to trigger the offset of the visual CS and initiate the delayed delivery (2.0 s) of a drop of juice reward, the total time required for the gaze to remain in the HA was ≥ 230 ms. If the gaze remained in the HA for < 230 ms, the visual CS was switched off and the animal had to continue searching. If the monkey failed to locate the HA within a search time of 30 s, the trial was terminated without reward and a new initial FP was presented. The inter-trial interval was 6.1–9.4 s for monkey U and 2.1–5.4 s for monkey Tb. During the session, when the search time appeared to be saturated within the range less than 7 s constantly through more than 80% for the successive 30 trials, it was judged as the learning was completed. Then, HA was then changed to another position in the screen. During each experimental day, the CS was presented consistently either in the intact or in the affected field throughout the sessions.

‘No CS’ condition: We instituted a blocks of trials in some sessions where everything was held constant except that CS presentation was omitted. During this block, even when monkey’s gaze entered the HA and remain in the HA for > 30 ms, a visual CS was not presented neither in the intact or in the affected field. However, if the total time that the gaze remained in the HA was ≥ 230 ms, a drop of juice reward was delivered after 2 s delay.

### Quantitative analysis of the time courses of behavioral acquisition in the instrumental conditioning task

The monkey’s search time “y” across trials within a session was fitted by a decaying exponential function:

$$ {\text{y}} = {\text{a}} + {\text{b}} \times {\text{exp}}\left( { - {\text{x}}/{\text{c}}} \right) $$

“a” is the asymptote, “a + b” is the value when x = 0, and “c” is the factor by which “y” (search time) changes with the number of trials (“x” increases). In combination, “a”, “b” and “c” parameters define different types of exponential curve that characterize the dynamics of learning the location of the HA and the systematic reduction of learning time course is represented as those coefficients (STable 2 and 3). The parameters of the exponential function were determined via a nonlinear least squares optimization method. The regression of all curves fitted in this way is statistically reliable by the root mean squared error (RMSE) and the goodness of fit (see STable 2 and 3).

### Analysis of the final saccade

To provide further insight into exactly what aspects of the animals’ instrumental behaviour were being conditioned, we performed a detailed analysis of the final saccade that located the HA. To do this we defined the onset of a saccade as the time point when the eye velocity exceeded 50°/s and later the movement’s peak velocity exceeded 100°/s. We selected 50°/s as a threshold to exclude the possibility of sampling slow eye movements such as smooth pursuit eye movements. A saccade offset was defined as the time point when the eye velocity slowed to < 50°/s. Final saccades were defined as the last saccade before a CS presentation for > 230 ms with the gaze remaining within the HA. Variance of direction of final saccades was calculated by the functions described below^51^:

$$ \begin{aligned} & {\text{Mean}}\,{\text{of}}\,{\text{vectors:}}\quad \left( {{\text{Rcos}}\theta ,{\text{Rsin}}\theta } \right) = {1}/{\text{N}}\left( {\Sigma {\text{cos}}\theta ,\Sigma {\text{sin}}\theta } \right) \\ & {\text{Variance}}\,{\text{of}}\,{\text{direction:}}\quad {\text{ V }} = { 1} - {\text{R}} \\ \end{aligned} $$

Mean of the vectors was calculated by averaging the summation of all the direction vectors (amplitude = 1), which, if the final saccade approached the HA equally from all directions, would have a length of 0. The length of the mean vector (R) therefore depends on variance of direction. Thus, the value V = 1 indicates a uniform dispersion from all directions, while and the value V = 0 means entry to the HA would be from a single direction.

### Analysis of distribution of saccade end points before and after the CS presentation

Similarity of the distributions of saccade end points during (*A*) and after (*B*) the search period was quantitatively assessed by modifying Ripley's *K*-functions^27^. In its modified version to compare the *A*-distribution with the *B*-distribution, we displaced the *A*-saccade end points to the Gaussian kernel and the analyzed field was divided by 2.5° squares. *A*-density of each square was defined as sum of closing Gaussian kernel value at the central point of each square. Each *B*-point was weighted by *A*-density of sequence on which the *B*-point landed. In this analysis, the homogeneity value was defined as the modified *KAB’(h)* (*mKAB’(h)*);

- *mKAB’(h)* = Sum of weighted value of square which *B*-point landed / *na* × *nb*
- *na*: number of *A*-points
- *nb*: number of *B*-points

With this analysis, similarity of distribution of saccade end points before and after the CS presentation (until the reward delivery) was evaluated between the sessions with CS in the intact field and those with CS in the affected field.

All methods are reported in accordance with ARRIVE (Animal Research: Reporting of in Vivo Experiments) guidelines^52^.

## Supplementary Information

Supplementary Information.

## Data Availability

The data analyzed during this study are available from corresponding authors upon reasonable request.
