# Supplementary material for: Visual instrumental learning in blindsight monkeys
Source: Sci Rep. 2021 Jul 20;11:14819. doi: 10.1038/s41598-021-94192-7 (PMC8292513; doi:10.1038/s41598-021-94192-7)
Supplement: Supplementary file 1 — Supplementary Information. [file 41598_2021_94192_MOESM1_ESM.docx]

Supplementary Information

**Visual instrumental learning in blindsight monkeys**

**Rikako Kato^1,2,6*^, Abdelhafid Zeghbib^3^, Peter Redgrave^3^, Tadashi Isa^1,2,4,5,6^**

**Supplementary Methods**

**Deficit maps**

To test sensitivities to the onset of luminance contrast, a target was presented at one of five possible locations in the hemi-visual field (SFig. 1). The stimuli radiated from the central FP with the same eccentricities Luminance contrast of the targets was varied randomly trial-by-trial (0.02 to 0.9 as expressed in Michelson contrast (Weber contrast 0.04-18.0)). The background luminance was set at 1.0 cd/m^2^ (gray background) for all tasks. Data of monkey U in SFig. 1 were reanalyzed from Fig.2A of Kato et al. (2011)^1^. Psychometric functions were constructed by plotting the correct ratio to the luminance contrast of the visual target. We calculated to fit a psychometric function with a cumulative Gaussian to the data. The luminance sensitivity was defined as a contrast value representing the percentage of correct responses corresponding to the sensitivity value d’ = 2 (threshold for luminance contrast). However, the deficit map of monkey Tb was once presented in Fig .3b of previous paper by Yoshida et al. (2008)^2^, in which threshold for luminance contrast in the ipsilesional (intact) field was all < 0.2. The contralesional (affected) visual field was reconstructed 8 months after the present experiments and shown here, indicating no improvement in the visual sensitivity.

**Criteria for selecting a learning session**

For population analysis, we selected the sessions that satisfied the following first screening criteria for further analysis;

1. The number of trials during the session >100 (58 sessions)
2. The mean search time of the first twenty trials > 5 s, the mean search time during the second half of the trials after Trial index < 7 s and the former was longer than the latter. Trial index : Coming down from the point of “-50ms/trial” on the fitting curve by a decaying exponential function (see methods), point to the trial number axis
3. The asymptote (‘a’) of the fitting curve by a decaying exponential function was over zero.

A total of 58 sessions were conducted with more than 100 trials in 2 monkeys (26 sessions for CS in the intact side and 32 sessions for CS in the lesion-affected side). Among these 23 sessions (40 %; 6 sessions for the intact side and 17 sessions for the affected side) were discarded from the analysis, because (i) discovery of the target location was too rapid (the mean search time of the first twenty trials ≤ 5 s – note that for the intact side and the lesion-affected side respectively, 4 out of the 6 excluded sessions and 12 out of the 17 excluded sessions were of this type); or (ii) the experimenter terminated the session before the target location was reliably discovered; the mean search time during the second half of the trials after the trial of Trial index was ≥ 7 s (2 of the excluded sessions on the intact side and 4 of the excluded sessions on the lesion-affected side were of this type). (iii) The asymptote (‘a’) of the fitting curve by a decaying exponential function was less than zero (1 of the excluded sessions on the lesion-affected side were of this type).

**Limitation of this study**

Quantitative analysis of the learning process in the sessions with CS presentations to the affected visual fields or the intact visual fields was made on the population data of 35 sessions (see Methods for the detail) (Fig. 3g and Fig.4g). However, in this study making quantitative comparisons of learning efficiency between CS presentation in intact vs lesion-affected visual fields could not be achieved. The reason is that the distribution of monkey’s spontaneous eye positions was biased to the left upper visual field in monkey U. This uncontrollable eye position bias would have affected the possibility of finding the HA by chance. In addition, the size of the target area and distance between the HAs in successive sessions were not completely randomized across sessions, because the eye movements during a search depended on the learning history. Before each experiment, it was difficult to predict the distribution of start positions of the last saccades that the monkeys would learn in the session. Therefore, quantitative comparison of learning process between the CS-in intact field and CS-in affected field conditions was not made in this study. These qualifications, however, do not undermine the main finding that instrumental conditioning can be effectively reinforced when the CS is presented to the lesion-affected visual field.

**Blinding**

We did not make a blinded experiment and analysis, because it was possible to determine the affected visual field in the V1 lesion monkeys by their behavior. To ensure the correctness of quantitative curve fitting analysis of the learning time courses by a decaying exponential function, the analysis was double checked by two people.

**Supplementary Figures**

**
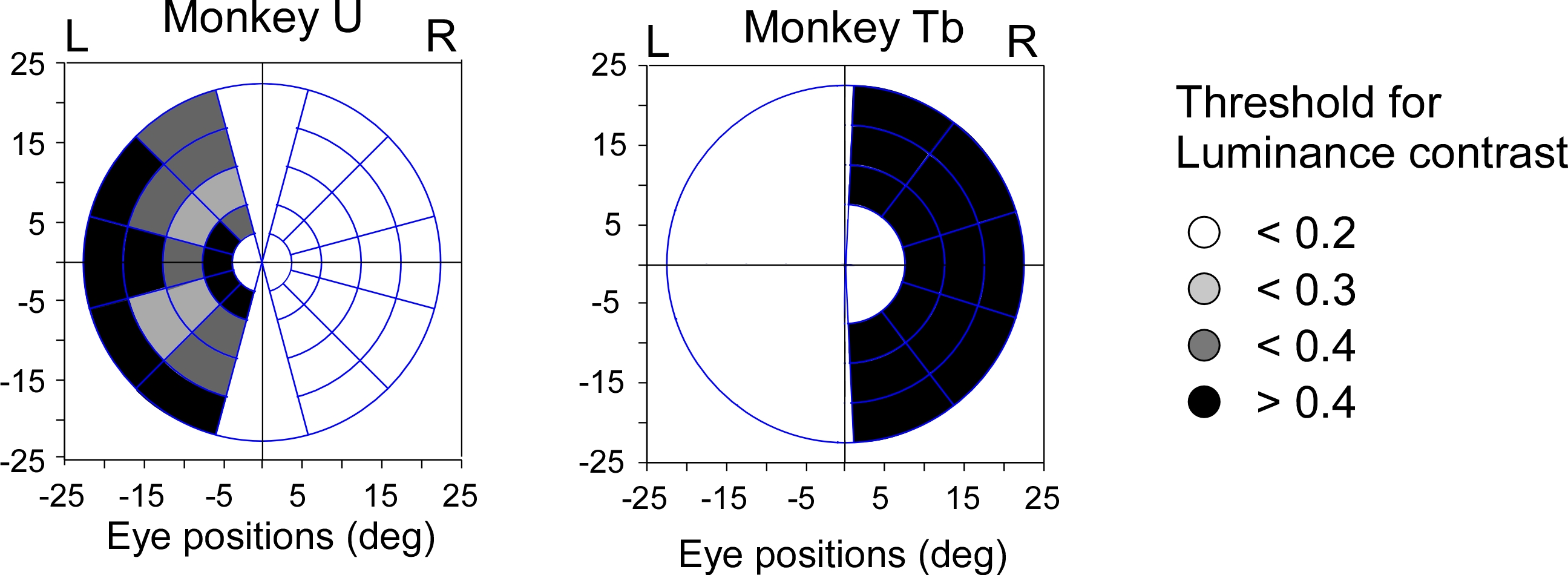
**

**SFigure 1.** Deficit maps of monkey U and monkey Tb. Sensitivities to luminance contrast (Michelson contrast) at various parts of the visual fields of two monkeys after V1 lesion. The thresholds for luminance contrast at individual target positions are displayed as a grayscale (see inset). Sensitivity to luminance contrast was clearly reduced widely in the lesion-affected visual field of both monkeys.

**Supplementary Tables**

**STable 1 List of experiments**

**STable 2　Exponential curve fitting of search times with CS in the intact visual field**

The monkey’s search time (ms) “y” across trials within a session was fitted by a decaying exponential function:

y = a + b × exp (-x/c)

HA: Hidden area, Size : diameter of HA, RMSE: root mean squared of error, R^2^: R-square

**STable 3 Exponential curve fitting of search times with CS in the affected visual field**

HA: Hidden area, Size : diameter of HA, RMSE: root mean squared of error, R^2^: R-square

**REFERENCES**

1. Kato, R., Takaura, K., Ikeda, T., Yoshida, M. & Isa, T. Contribution of the retino-tectal pathway to visually guided saccades after lesion of the primary visual cortex in monkeys. *Eur. J. Neurosci.* **33**, 1952–1960 (2011).

2. Yoshida, M., Takaura, K., Kato, R., Ikeda, T. & Isa, T. Striate cortical lesions affect deliberate decision and control of saccade: Implication for blindsight. *J. Neurosci.* **28**, 10517–10530 (2008). exponential fitting
